# Supplementary material for: Gapless assembly of maize chromosomes using long-read technologies
Source: Genome Biol. 2020 May 20;21:121. doi: 10.1186/s13059-020-02029-9 (PMC7238635; doi:10.1186/s13059-020-02029-9)
Supplement: Supplementary file 1 — Additional file 1: Figure S1. Workflow for the B73-Ab10 assembly pipeline. Figure S2. Complementation of PacBio assembly gaps by Nanopore contigs. Figure S3. The alignment of BAC-based assemblies of B73 centromeres to the merged assembly in optical map format. Table S1. Assembly statistics and gaps in B73-Ab10 assemblies. Table S2. Accuracy of genome assemblies as assessed by comparison to Bionano maps. Table S3. Coordinates and composition of centromeres defined by CENH3 ChIP-seq in the B73-Ab10 assembly. Table S4. CENH3 enrichment and mappability of Illumina reads in active centromeres. Table S5. Repetitive components in B73-Ab10 assemblies. Table S6. Composition of CentC arrays. Table S7. Composition of knob180 and TR-1 knobs. Table S8. Gene and transposon distributions in the Ab10 haplotype and corresponding N10 regions. [file 13059_2020_2029_MOESM1_ESM.docx]

**
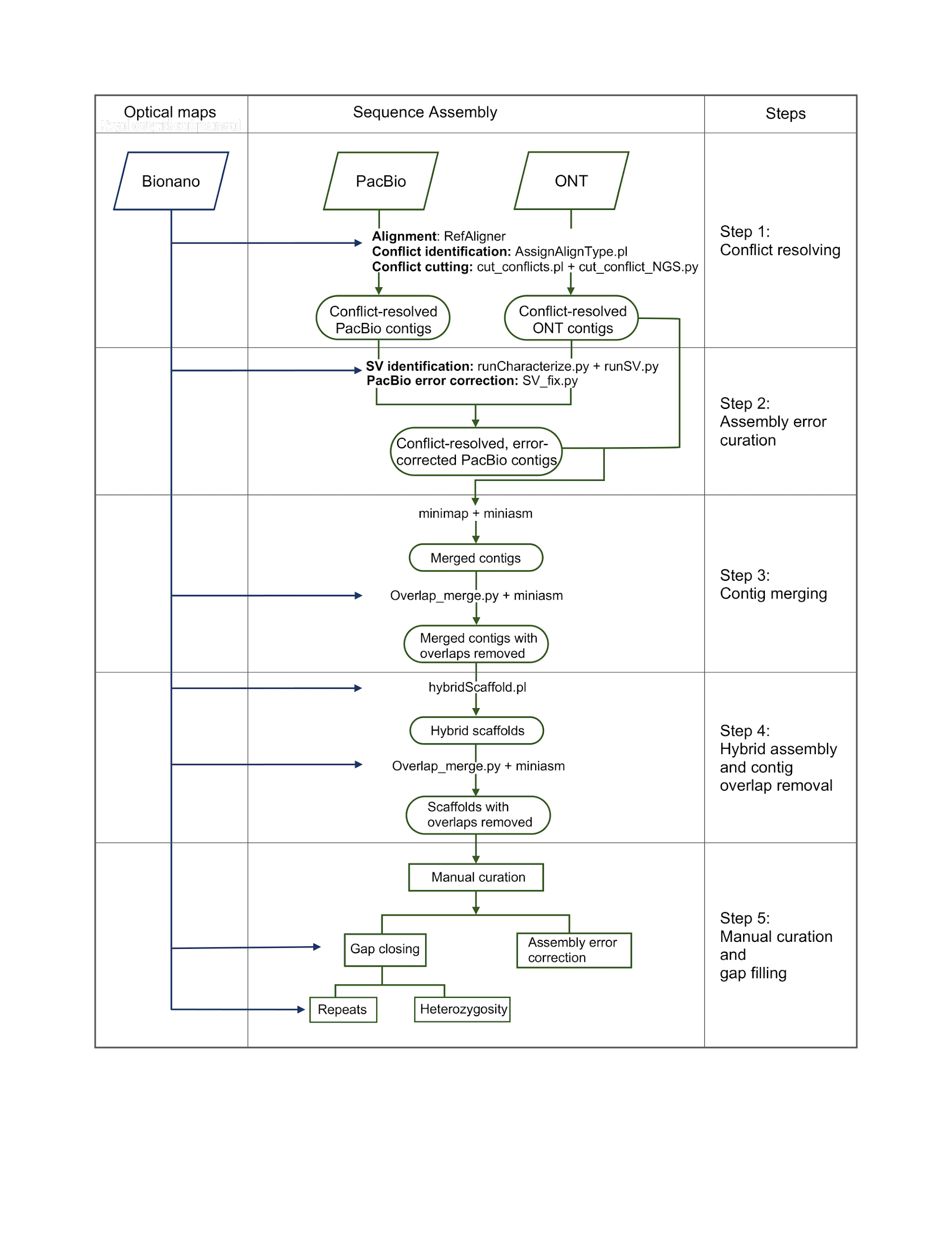
**

**Fig S1.** Workflow for the B73-Ab10 assembly pipeline.

**Fig S2**. Complementation of PacBio assembly gaps by Nanopore contigs. **A**) Co-occurrence of PacBio assembly gaps with tandem repeats and heterozygosity. Tandem repeat arrays were defined as having at least 25 Kb of tandem repetitiveness with a maximum interval of 300 Kb between repeat units. The table illustrates that the majority of PacBio assembly gaps, either from contig overlaps (indicated by 13N) or gaps of known size, were complemented by ONT contigs. The correlations between PacBio assembly gaps and tandem repeat arrays or heterozygous regions are also shown (where P values were calculated by Fisher’s exact test). The boxplot on the right shows the size distribution of repeat arrays (with at least 25 tandem repeats) that were fully assembled by PacBio data, those that were not assembled but successfully complemented by ONT data, and those associated with gaps that remain in the final assembly. The tandem repeat highlighted with a triangle is displayed in B. **B**) Example of a large tandem repeat region on chr8: 31-33.5 Mb. PacBio and ONT read alignment (MAPQ>=0) is shown as a heatmap with 10 Kb windows. Read gaps were defined as regions with fewer than three reads for PacBio error-corrected reads and Illumina reads, and fewer than two reads for ONT error-corrected dataset. Tracks showing no information have no gaps. Length distributions of PacBio and ONT reads mapped to the corresponding region are displayed in a histogram on the right. **C**) Example of a large heterozygous region on chr3: 164-167.6 Mb. Tracks are annotated as in B. Length distributions of PacBio and ONT reads mapped to the corresponding region are displayed in a histogram on the right. **D**) Whole chromosome view of assembly and read mapping on chr8.

**
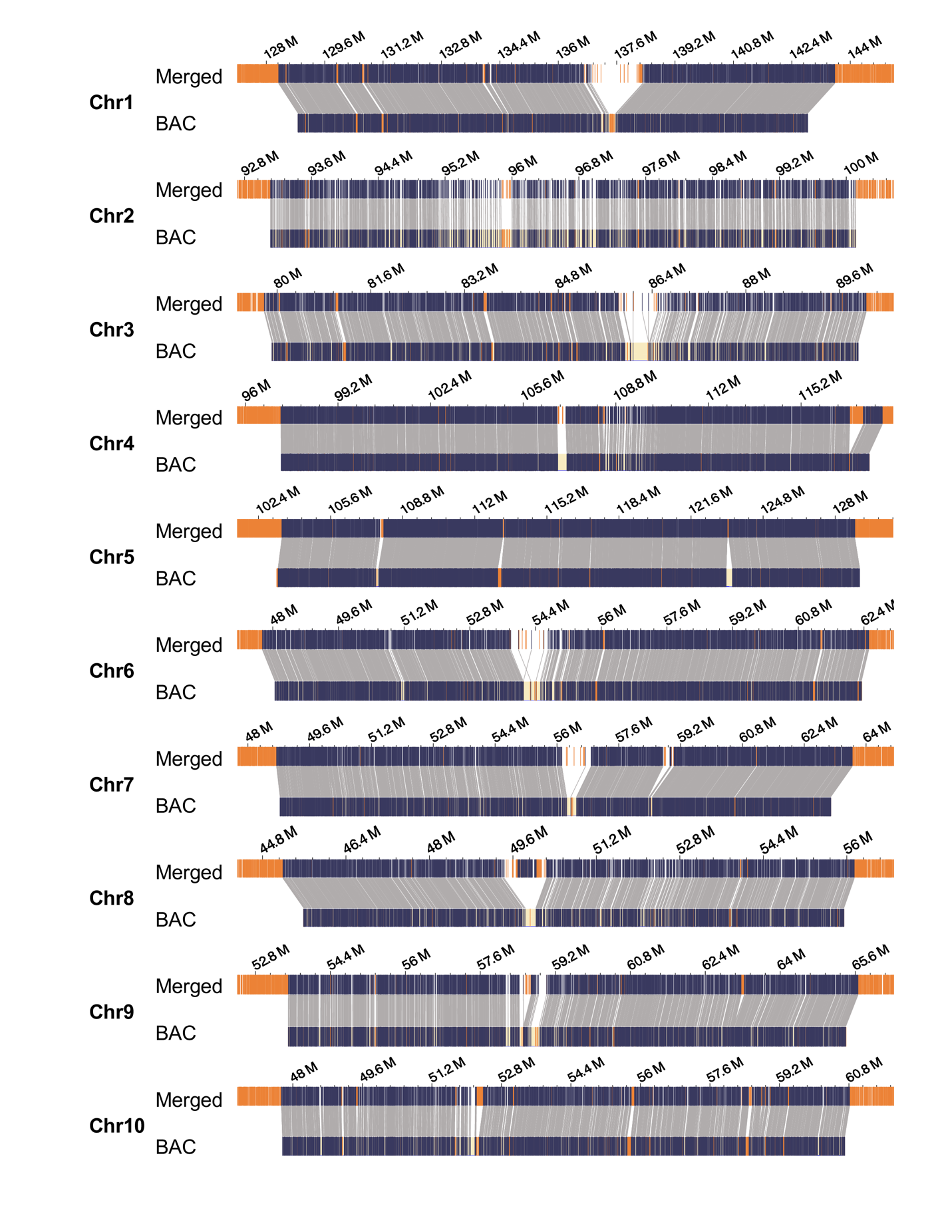
**

**Fig S3.** The alignment of BAC-based assemblies of B73 centromeres to the merged assembly in optical map format. The connecting lines represent matching regions between the two assemblies.

|  | Metrics | Nanopore | PacBio | Merged |
| --- | --- | --- | --- | --- |
| Contig | N50 (Mb) / L50 | 2.0 / 325 | 41.2 / 16 | 162.0 / 6 |
|  | N60 (Mb) / L60 | 1.7 / 440 | 31.5 / 22 | 111.4 / 8 |
|  | N70 (Mb) / L70 | 1.3 / 584 | 23.8 / 30 | 88.8 / 10 |
|  | N80 (Mb) / L80 | 0.9 / 775 | 15.2 / 42 | 50.1 / 13 |
|  | N90 (Mb) / L90 | 0.6 / 1061 | 7.4 / 64 | 20.4 / 21 |
|  | Total contig number | 1912 | 1103 | 1016 |
|  | Total sequence (Mb) | 2120.5 | 2232.9 | 2241.4 |
|  | Contig number in Bionano scaffolds ^a^ | 1699 | 211 | 132 ^d^ |
|  | Contig sequence in scaffolds (Mb) ^a^ | 2072.5 | 2156.5 | 2174.2 |
| Scaffold | Number | 43 | 25 | 50 |
|  | N50 (Mb) | 125.7 | 162.9 | 161.8 ^e^ |
|  | Max (Mb) | 201.2 | 236.5 | 235.9 |
|  | Total length (Mb) | 2170.1 | 2162.2 | 2178.1 |
|  | Contig overlaps ^b^ | 929 | 114 | 12 |
|  | Gaps of known size ^c^ | 728 | 73 | 51 |
|  | Total known gap size (Mb) | 95.1 | 2.6 | 2.3 |
| Pseudo-  molecules | LTR Assembly Index (LAI) | 8.57 | 27.98 | 27.8 ^f^ |
|  | BUSCO (% Complete) | 90.7 | 95.6 | 95.8 |
|  | Total length (Mb) | 2161.1 | 2162.7 | 2162.8 |
|  | Contig overlaps ^b^ | 910 | 114 | 5 |
|  | Gaps of unknown size | 30 | 16 | 17 |
|  | Gaps of known size ^c^ | 728 | 73 | 31 |
|  | Total known gap size (Mb) | 93.2 | 2.6 | 1.3 |
|  | Assembled telomeres | 1 | 9 | 15 |

**Table S1. Assembly statistics and gaps in B73-Ab10 assemblies.**

^a^ Contigs included in scaffolds are conflict-resolved contigs after hybrid assembly.

^b^ Contig overlaps are identified when the contigs are integrated with the optical map. The Bionano hybrid scaffolding software marks them with 13 Ns.

^c^ Gap sizes are estimated by Bionano maps during hybrid assembly.

^d^ Only 63 are included in pseudomolecules; the remaining 59 are anchored to small scaffolds (< 3Mb) containing CentC or knob arrays that lack genetic and pan-genome markers and could not be placed on chromosomes (Suppl. Table 5).

^e^ The N50 is smaller in Merged than PacBio due to the correction of the CentC array on chromosome 9 (Figure 1B).

^f^ LAI is lower in the Merged than PacBio due to a reduction in the total number of LTRs, presumably because sequence overlaps were removed.

**Table S2. Accuracy of genome assemblies as assessed by comparison to Bionano maps.**

|  | Nanopore | PacBio | Merged |
| --- | --- | --- | --- |
| Contig misjoins / Conflict cuts | 425 | 18 | 1 |
| Number of collapsed repeats > 25 Kb | 705 | 56 | 3 |
| Sequence lost by collapse of repeats > 25 Kb (Mb) | 44.80 | 3.65 | 0.13 |
| Number of expanded repeats > 25 Kb | 22 | 13 | 10 |
| Sequence gained by expansion of repeats > 25 Kb (Mb) | 1.28 | 1.49 | 0.57 |

**Table S3. Coordinates and composition of centromeres defined by CENH3 ChIP-seq in the B73-Ab10 assembly.**

| Chr | Start (bp) | End (bp) | Size (bp) | 100N^a^ | CentC (%) | CRM (%) | cinful-zeon (%) | grande (%) | huck (%) | opie-ji (%) | prem1 (%) |
| --- | --- | --- | --- | --- | --- | --- | --- | --- | --- | --- | --- |
| chr1 | 137,090,000 | 138,130,000 | 1,040,000 | Y | 54.8 | 41.1 | 0 | 0 | 0 | 0.9 | 0.1 |
| chr2 | 95,290,000 | 97,165,000 | 1,875,000 | N | 1.3 | 31.5 | 14 | 3.2 | 6.3 | 2.5 | 7.6 |
| chr3 | 85,880,000 | 87,705,000 | 1,825,000 | N | 14.7 | 37.2 | 8.8 | 0 | 4.8 | 2 | 4.6 |
| chr4 | 108,485,000 | 110,135,000 | 1,650,000 | N | 1 | 44.6 | 10.2 | 0.5 | 3.6 | 3.5 | 6.3 |
| chr5 | 104,255,000 | 106,220,000 | 1,965,000 | N | 0 | 16.3 | 19.2 | 0.8 | 2.2 | 1.5 | 8.1 |
| chr6 | 53,825,000 | 54,655,000 | 830,000 | Y | 49.1 | 41.4 | 0.8 | 0 | 1.5 | 0.2 | 0.0 |
| chr7 | 56,220,000 | 56,860,000 | 640,000 | Y | 30.8 | 54.5 | 0.3 | 0 | 0 | 0 | 0.0 |
| chr8 | 51,095,000 | 52,795,000 | 1,700,000 | N | 0 | 25.3 | 17.9 | 3.9 | 1.5 | 1.3 | 4.3 |
| chr9 | 54,840,000 | 56,275,000 | 1,435,000 | N | 0 | 5.1 | 18.8 | 6.4 | 2.8 | 2.2 | 11.1 |
| chr10 | 50,845,000 | 52,505,000 | 1,660,000 | N | 9.3 | 22.3 | 13.1 | 1.8 | 3.2 | 5.2 | 7.1 |

^a^ Gaps marked by 100 Ns are of unknown size.

**Table S4. CENH3 enrichment and mappability of Illumina reads in active centromeres.**

| Chr | Mappability^a^ | CentC  length (kb) / fold change ^b^ | CRM  length (kb) / fold change | cinful-zeon  length (kb) / fold change | grande length (kb) / fold change | huck  length (kb) / fold change | opie-ji  length (kb) / fold change | prem1  length (kb) / fold change |
| --- | --- | --- | --- | --- | --- | --- | --- | --- |
| chr1 | Unique | 339.3 / 10.0 | 59.7 / 7.3 | 0 / NA | 0 / NA | 0 / NA | 9.3 / 8.6 | 0 / NA |
|  | Non-unique | 226.5 / 1.7 | 390.6 / 4.3 | 0 / NA | 0 / NA | 0 / NA | 0 / NA | 0 / NA |
| chr2 | Unique | 24.9 / 32.7 | 167.2 / 18.2 | 229.1 / 16.0 | 24.5 / 19.2 | 113.0 / 11.6 | 27.4 / 20.6 | 54.3 / 21.4 |
|  | Non-unique | 0 / NA | 470.0 / 8.4 | 51.7 / 8.1 | 36.0 / 6.0 | 0 / NA | 23.9 / 4.4 | 12.7 / 4.2 |
| chr3 | Unique | 213.5 / 15.1 | 166.5 / 12.2 | 121.4 / 14.6 | 0 / NA | 61.5 / 7.4 | 23.7 / 11.7 | 25.1 / 15.6 |
|  | Non-unique | 53.8 / 1.3 | 598.1 / 7.8 | 38.6 / 9.1 | 0 / NA | 27.0 / 1.1 | 18.3 / 2.2 | 8.5 / 7.1 |
| chr4 | Unique | 17.1 / 23.6 | 178.3 / 14.0 | 138.4 / 17.7 | 4.7 / 42.5 | 59.3 / 7.3 | 51.1 / 16.2 | 52.4 / 19.1 |
|  | Non-unique | 0 / NA | 622.5 / 9.1 | 45.9 / 5.7 | 0 / NA | 0 / NA | 33.6 / 5.7 | 0 / NA |
| chr5 | Unique | 0 / NA | 26.5 / 10.1 | 354.3 / 20.3 | 15.0 / 10.0 | 27.4 / 3.1 | 32.0 / 18.1 | 132.1 / 18.2 |
|  | Non-unique | 0 / NA | 321.7 / 6.6 | 33.4 / 4.0 | 0 / NA | 15.6 / 2.3 | 0 / NA | 0 / NA |
| chr6 | Unique | 242.4 / 11.0 | 57.7 / 11.4 | 6.3 / 5.5 | 0 / NA | 12.4 / 2.9 | 1.8 / 21.8 | 0 / NA |
|  | Non-unique | 162.8 / 1.8 | 313.0 / 5.8 | 0 / NA | 0 / NA | 0 / NA | 0 / NA | 0 / NA |
| chr7 | Unique | 101.2 / 10.4 | 66.2 / 6.3 | 2.1 / 18.5 | 0 / NA | 0 / NA | 1.5 / 23.2 | 0 / NA |
|  | Non-unique | 95.0 / 2.4 | 317.1 / 5.4 | 0 / NA | 0 / NA | 0 / NA | 0 / NA | 0 / NA |
| chr8 | Unique | 0 / NA | 275.8 / 17.8 | 54.0 / 19.8 | 65.4 / 18.2 | 25.6 / 22.7 | 26.9 / 22.3 | 53.0 / 19.7 |
|  | Non-unique | 0 / NA | 49.4 / 4.7 | 418.0 / 7.4 | 0 / NA | 0 / NA | 0 / NA | 16.6 / 12.7 |
| chr9 | Unique | 0 / NA | 24.5 / 33.3 | 227.4 / 19.6 | 89.0 / 27.3 | 40.9 / 16.3 | 31.6 / 29.4 | 102.0 / 20.2 |
|  | Non-unique | 0 / NA | 73.6 / 15.6 | 46.4 / 4.2 | 0 / NA | 0 / NA | 9.0 / 9.9 | 29.8 / 10.3 |
| chr10 | Unique | 119.6 / 13.5 | 140.2 / 16.5 | 196.1 / 22.8 | 19.3 / 40.0 | 52.5 / 7.2 | 73.3 / 18.0 | 76.3 / 22.5 |
|  | Non-unique | 33.4 / 1.5 | 264.7 / 7.6 | 33.3 / 4.7 | 8.6 / 4.6 | 1.5 / 11.6 | 34.3 / 6.5 | 0 / NA |
| Total | Unique | 1058.1 / 12.4 | 940.7 / 18.7 | 1550.9 / 14.4 | 218.0 / 23.9 | 392.7 / 10.6 | 257.8 / 18.8 | 495.1 / 19.8 |
|  | Non-unique | 571.4 / 1.8 | 3798.1 / 5.9 | 298.7 / 7.4 | 44.6 / 5.7 | 44.1 / 1.9 | 86.2 / 5.4 | 67.7 / 9.3 |

^a^ Non-unique regions are defined as sequences with too low (<3) or low high (>100) numbers of mapped Illumina reads. The total genome coverage was 30X.

^b^  Fold change is expressed as the ratio of CENH3 ChIP-seq reads to genomic reads for each repeat type.

**Table S5. Repetitive components in B73-Ab10 assemblies.**

|  | Repeat Type | Nanopore | PacBio | Merged |
| --- | --- | --- | --- | --- |
| Scaffold | knob180 (bp) | 3,962,210 | 9,893,818 | 16,861,684 |
|  | TR-1 (bp) | 2,324,807 | 4,984,957 | 7,253,994 |
|  | CentC (bp) | 708,654 | 3,233,366 | 2,881,960 |
|  | rDNA intergenic spacer (bp) | 185,738 | 789,045 | 755,420 |
|  | Subtelomere (bp) | 99,243 | 504,890 | 563,144 |
| Pseudo-molecules | knob180 (bp) | 3,469,793 | 9,894,164 | 10,371,693 |
|  | TR-1 (bp) | 1,824,356 | 4,984,826 | 4,979,336 |
|  | CentC (bp) | 708,574 | 3,233,759 | 2,939,574 |
|  | rDNA intergenic spacer (bp) | 185,513 | 789,045 | 622,630 |
|  | Subtelomere (bp) | 99,218 | 554,866 | 598,330 |

**Table S6. Composition of CentC arrays.**

| Chr | Start (bp) | End (bp) | Size (bp) | 100N^a^ | CentC (%) | CRM (%) | cinful-zeon (%) | grande (%) | huck (%) | opie-ji (%) | prem1 (%) |
| --- | --- | --- | --- | --- | --- | --- | --- | --- | --- | --- | --- |
| chr1 | 133,797,033 | 134,161,088 | 364,055 | N | 28.2 | 1.9 | 9.0 | 3.8 | 6.2 | 2.6 | 3.7 |
| chr1 | 136,702,219 | 138,370,323 | 1,668,104 | Y | 45.7 | 38.8 | 2.4 | 0.0 | 1.5 | 0.9 | 0.6 |
| chr2 | 95,886,484 | 96,018,046 | 131,562 | N | 19.1 | 45.8 | 10.7 | 0.0 | 0.0 | 6.8 | 6.5 |
| chr3 | 83,463,110 | 83,579,253 | 116,143 | N | 29.9 | 0.0 | 6.4 | 12.3 | 11.2 | 0.0 | 6.8 |
| chr3 | 85,837,492 | 86,608,170 | 770,678 | N | 37.8 | 41.3 | 3.8 | 0.0 | 3.7 | 1.1 | 3.3 |
| chr4 | 109,241,724 | 109,363,707 | 121,983 | N | 14.2 | 61.3 | 4.0 | 0.0 | 0.0 | 0.0 | 0.0 |
| chr5 | 107,727,923 | 107,946,850 | 218,927 | N | 41.8 | 34.3 | 4.5 | 0.0 | 0.0 | 0.0 | 3.1 |
| chr6 | 53,802,144 | 54,696,183 | 894,039 | Y | 50.5 | 40.8 | 0.7 | 0.0 | 1.4 | 0.2 | 0.0 |
| chr7 | 56,131,392 | 56,883,231 | 751,840 | Y | 32.6 | 54.5 | 0.3 | 0.0 | 0.0 | 0.0 | 0.0 |
| chr7 | 58,725,452 | 59,026,217 | 300,765 | N | 73.7 | 2.3 | 0.0 | 0.0 | 0.0 | 6.1 | 5.3 |
| chr8 | 49,443,032 | 49,660,040 | 217,008 | N | 60.9 | 23.8 | 3.4 | 0.0 | 0.0 | 0.0 | 3.7 |
| chr8 | 50,101,170 | 50,244,111 | 142,941 | N | 47.8 | 28.6 | 9.9 | 0.0 | 0.0 | 0.0 | 0.0 |
| chr9 | 58,430,781 | 58,672,011 | 241,230 | N | 59.7 | 15.8 | 0.0 | 0.0 | 0.0 | 0.0 | 8.5 |
| chr9 | 58,867,400 | 59002849 | 135,449 | N | 61.2 | 31.0 | 0.4 | 0.0 | 0.0 | 0.0 | 0.0 |
| chr10 | 49,448,433 | 49,559,148 | 110,715 | N | 67.8 | 0.0 | 0.0 | 0.0 | 11.1 | 0.0 | 8.6 |
| chr10 | 52,128,660 | 52,443,218 | 314,558 | N | 49.2 | 31.1 | 5.2 | 0.0 | 0.0 | 4.2 | 3.2 |

^a^ Gaps marked by 100 Ns are of unknown size.

**Table S7. Composition of knob180 and TR-1 knobs.**

|  | Chr | Start (bp) | End (bp) | Size (bp) | 100N^a^ | Ngap (%) | knob180 (%) | TR-1 (%) | cinful-zeon (%) | grande (%) | huck (%) | opie-ji (%) | prem1 (%) |
| --- | --- | --- | --- | --- | --- | --- | --- | --- | --- | --- | --- | --- | --- |
| TR-1 | chr4 | 231,661,938 | 232,984,750 | 1,322,813 | N | 0.0 | 0.1 | 51.0 | 30.3 | 0.0 | 0.0 | 1.9 | 1.9 |
|  | chr10 | 142,321,698 | 146,554,927 | 4,233,230 | N | 0.0 | 1.1 | 42.7 | 27.3 | 0.7 | 1.2 | 1.2 | 3.1 |
|  | chr10 | 150,506,817 | 153,088,305 | 2,581,489 | N | 0.0 | 0.0 | 42.0 | 29.7 | 1.2 | 1.7 | 1.4 | 2.2 |
|  | chr10 | 157,208,255 | 159,276,069 | 2,067,815 | N | 0.0 | 0.5 | 36.9 | 20.9 | 0.6 | 2.5 | 4.9 | 5.9 |
| knob180 | chr5 | 198,844,996 | 200,124,258 | 1,279,263 | N | 0.0 | 59.3 | 5.0 | 12.0 | 1.1 | 1.1 | 2.0 | 5.0 |
|  | chr6 | 1 | 623,495 | 623,495 | N | 14.9 | 56.7 | 2.3 | 4.7 | 2.3 | 1.9 | 0.0 | 4.5 |
|  | chr6 | 176,451,347 | 177,079,220 | 627,874 | N | 0.0 | 28.9 | 18.4 | 11.9 | 4.5 | 1.5 | 4.0 | 0.8 |
|  | chr7 | 155,073,718 | 157,644,910 | 2,571,193 | Y | 7.0 | 66.0 | 2.7 | 5.9 | 0.0 | 0.6 | 2.8 | 1.6 |
|  | chr8 | 160,828,702 | 162,735,401 | 1,906,700 | Y | 15.0 | 58.2 | 3.7 | 6.3 | 0.0 | 0.7 | 1.4 | 1.6 |
|  | chr9 | 1,630 | 842,889 | 841,260 | N | 0.0 | 67.0 | 4.3 | 7.4 | 1.7 | 5.9 | 4.3 | 0.9 |
|  | chr10 | 174,217,005 | 178,146,998 | 3,929,994 | Y | 7.8 | 63.6 | 0.8 | 7.6 | 0.4 | 1.7 | 2.0 | 1.5 |
|  | chr10 | 180,357,409 | 182,945,132 | 2,587,724 | N | 0.0 | 53.9 | 0.0 | 9.8 | 1.1 | 0.5 | 5.6 | 4.0 |

^a^ Gaps marked by 100 Ns are of unknown size.

**Table S8. Gene and transposon distributions in the Ab10 haplotype and corresponding N10 regions.**

| Region | N10/Ab10 Proximal ^a^ | Ab10 Shared ^b^ | Ab10 Specific ^c^ |
| --- | --- | --- | --- |
| Size (bp) | 20,000,000 | 12,716,384 | 22,438,721 |
| Genes | 521 | 580 | 450 |
| Gene density (genes/Mb) | 26.1 | 45.6 | 20.1 |
| CDS content (%) | 8.8 | 15.0 | 5.5 |
| Average gene length (bp) | 3,377 | 3,284 | 2,757 |
| Average CDS length (bp) | 1,465 | 1,425 | 911 |
| Single exon gene (%) | 35.5 | 36.0 | 49.1 |
| Genes overlapped with TE by 95% (%) | 4.2 | 4.3 | 7.9 |
| TE content (%) | 74.2 | 76.4 | 88.1 |

^a^ Sequence in a 20 Mb region left of the first TR-1 knob that is not a part of the Ab10 haplotype (122.3 - 142.3 Mb).

^b^ Sequence in two large inversions with shared synteny between the Ab10 haplotype and N10 (153.0 -157.4 Mb and 159.4 - 167.7 Mb).

^c^ Sequence present in the Ab10 haplotype but not the B73 N10 genome, including a region between the first two TR-1 knobs (146.6 - 150.5 Mb), a region from the end of the second inversion to the large knob (167.7 - 174.3 Mb), and a region from the large knob to the end of Ab10 haplotype (183.1 - 195.0 Mb).
