# Supplementary material for: Gapless assembly of maize chromosomes using long-read technologies
Source: Genome Biol. 2020 May 20;21:121. doi: 10.1186/s13059-020-02029-9 (PMC7238635; doi:10.1186/s13059-020-02029-9)
Supplement: Supplementary file 2 — Additional file 2. Review history. [file 13059_2020_2029_MOESM2_ESM.docx]

Review History

**First round of review**

**Reviewer 1**

**Are you able to assess all statistics in the manuscript, including the appropriateness of statistical tests used?**

There are no statistics in the manuscript.

**Comments to author:**

Liu et al. developed a novel assembly merging pipeline (leveraging both optical maps and long read sequence data from PacBio and ONT), which raised contiguity to a level well above any prior genome assembly. In doing so, the authors present the first assembly of gapless telomere-totelomere plant chromosomes, including seven centromeres and the inner structure of maize knobs. This is an extremely high-quality assembly product that should have broad interest in the genomics community and represents a significant advance over previously published studies. The methods described are appropriate to the aims of the study and are well described.  The conclusions presented here are supported by the data.  I have listed the following comments below as minor suggestions to help improve the manuscript:

1. It is unclear why the authors ran two different long read assemblers on the PacBio and ONT data.  Statements like "…where the superior PacBio assembly provides the core and Nanopore contigs help to seal the gaps" (L51), make it difficult for readers to understand if the assembly differences are due to the algorithm or the underlying data.
2. I understand that this work is presented as a short note, but I could not help but want more biological results. I understand that the authors did not provide BAC confirmation across the centromeres to provide a comprehensive validation of the structure, nevertheless I think it may increase the impact of the work to have a single figure dedicated to the centromere assembly work — showing what sequences are there, which are enriched with CENPC, and where one has the most support for structure.
3. Polishing of repeats is a very active area of research, it was unclear if the authors were only able to polish the regions of the genome that allowed short read unique mapping. (It appears that way based on L287-8).  Therefore the large tandem repeats that are in the assembly may have a higher error and may confound the precise mapping of CENH3 ChIP-seq.
4. These technologies move very fast, and it may be important to include more information on the nanopore sequencing (ie. did you use R9 or R10 flow cells, what base caller was used, what is the N50 read length).
5. Is it correct in my reading, that all assembled BioNano-guided assemblies were confidently assigned to a single chromosome?  I am trying to understand how many unknowns remain, if any.

**Reviewer 2**

**Are you able to assess all statistics in the manuscript, including the appropriateness of statistical tests used?**

There are no statistics in the manuscript.

**Comments to author:**

In this manuscript, Liu et al report a pipeline that merge two long read technologies and an optical map to produce a gapless assembly of maize chromosome. The data also present the internal structure of major tandem repeats including centromere and heterochromatic knob. I especially look forward to its potential usage in assembling complex genomes with polyploidy or higher levels of heterozygosity.
I have one major concern: I noticed that the only chromosome 3 and 9 are assembled with gapless. Why these two chromosomes are assembled with gapless, rather than other chromosomes? As they are not the shortest chromosome and they are not the chromosome with minimal satellite repeats in maize. Furthermore, did you use your pipeline with the public dataset from maize B73 to assembly the genome. It may be used to evaluate your pipeline with another repeat experiment.
Two minor questions:
1、The author reported that the Cinful-Zeon family of Gypsy elements preferentially target knobs, so how do your think the function of Cinful-Zeon elements in knobs?

2、The author provided the first complete assembly of mazie Abnomal chromosome 10. The kinesin driver complex binding to knob180 is required for meiotic drive. Whether TR-1 knobs have its own  motors encoded on Ab10?

**Authors Response**

**Point-by-point responses to the reviewers’ comments:**

Comment

*Response*

Reviewer #1:

Liu et al. developed a novel assembly merging pipeline (leveraging both optical maps and long read sequence data from PacBio and ONT), which raised contiguity to a level well above any prior genome assembly. In doing so, the authors present the first assembly of gapless telomere-totelomere plant chromosomes, including seven centromeres and the inner structure of maize knobs. This is an extremely high-quality assembly product that should have broad interest in the genomics community and represents a significant advance over previously published studies. The methods described are appropriate to the aims of the study and are well described. The conclusions presented here are supported by the data. I have listed the following comments below as minor suggestions to help improve the manuscript:

1. It is unclear why the authors ran two different long read assemblers on the PacBio and ONT data. Statements like "…where the superior PacBio assembly provides the core and Nanopore contigs help to seal the gaps" (L51), make it difficult for readers to understand if the assembly differences are due to the algorithm or the underlying data.

*Answer:
We used different long-read assemblies because at the time these were the best assemblers for the data generated. Canu had been optimized for maize PacBio data, whereas there had been no prior work with Nanopore data. It is likely that the use of different assemblers was a strength, since the assemblies proved to complement each other. We will not be able to rerun the assemblies using different approaches, however we have provided new data to help readers make their own inferences to guide future work.

The major factor is that the longer reads from the Nanopore data are more likely to traverse long repeat structures. We now present a new Supplementary Figure 2 showing this relationship as it relates to tandem repeats and regions of heterozygosity. While the PacBio assembly failed in most large repetitive and heterozygous regions, the Nanopore often succeeded in these areas (Supplementary Fig. 2A). Through mapping reads to the final genome, we found that PacBio reads often failed to align uniquely in large tandem repetitive regions, but a uniform mapping pattern was observed for nanopore data (Supplementary Fig. 2B). Therefore, we observed fragmented or absent PacBio assemblies over repeat regions but accurate genome sequence in the Nanopore assembly. Similarly, in heterozygous regions, long reads in Nanopore data were able to span junctions and help identify dominant paths, generating contiguous contigs (Supplementary Fig. 2C). The alternative paths could not be resolved with relatively shorter reads in PacBio dataset (Supplementary Fig. 2C), which prematurely aborted the unitigging process and produced overlapping fragmented contigs.

Another factor may be that Canu uses error-corrected PacBio reads for assembly, while miniasm uses uncorrected Nanopore reads. The error correction process that is necessary for PacBio assembly may have homogenized some repeats and limited the assembly in long repeat arrays. We have now pointed out that this might be a contributing factor in the appropriate methods.*

2. I understand that this work is presented as a short note, but I could not help but want more biological results. I understand that the authors did not provide BAC confirmation across the centromeres to provide a comprehensive validation of the structure, nevertheless I think it may increase the impact of the work to have a single figure dedicated to the centromere assembly work — showing what sequences are there (that is table 3), which are enriched with CENPC, (add a row at showing enrichment over each repeat type) and where one has the most support for structure.

*Answer:
We appreciate this encouragement and advice. As suggested, in the revised manuscript we have added a new table showing CENH3 ChIP enrichment for each of the major sequence components (Supplementary Table 4). The results reveal that the sequence content and CENH3 enrichment for each repeat element are highly variable among chromosomes. The same table also addresses the question of structure (sequence diversity). While CentC is highly repetitive, 65% of the assembled CentC could be uniquely mapped by short reads. Similar trends were found for all repeats, and demonstrate that both unique and repetitive sequences can be bound by CENH3 and support centromere formation. These new results are now summarized in the main body of the paper. In addition, as recommended by the reviewer, we have added a ChIP-seq track to the inset of Figure 1A that illustrates where the active centromere lies over different types of repeats.*

3. Polishing of repeats is a very active area of research, it was unclear if the authors were only able to polish the regions of the genome that allowed short read unique mapping. (It appears that way based on L287-8). Therefore the large tandem repeats that are in the assembly may have a higher error and may confound the precise mapping of CENH3 ChIP-seq.

*Answer:
Polishing was performed at multiple stages. At each stage, whether it involved PacBio reads, Nanopore reads, or Illumina reads, the software required unique mapping. As described above, in highly repetitive regions, PacBio and Illumina reads often map to incorrect locations (Supplementary Figure 2B). Long Nanopore reads are more likely to map to repetitive and heterozygous regions, however, the coverage in these areas is low. Regions with too many reads are likely to be overpolished and regions with few reads will have a higher error rate. This is now clearly pointed out in the methods. The effects on CENH3 ChIP-seq read mapping appear to be minimal since the majority of reads in centromeres map uniquely.*

4. These technologies move very fast, and it may be important to include more information on the nanopore sequencing (ie. did you use R9 or R10 flow cells, what base caller was used, what is the N50 read length).

*Answer:
This information has been added to the Methods (Flow cells R9.4, Reads called using guppy (v2.1.3), N50 of read length is 29,311 bp)).*

5. Is it correct in my reading, that all assembled BioNano-guided assemblies were confidently assigned to a single chromosome? I am trying to understand how many unknowns remain, if any.

*Answer:
Upon hybrid scaffolding and manual curation, 26 of the 50 Bionano scaffolds with uniquely mapped genetic markers were included in the pseudomolecules. Among the 24 unplaced scaffolds with a total size of 19.4 Mb, 22 are composed entirely of knob180 and/or TR-1 arrays (17.7 Mb). This information is now included in the Methods.*

Reviewer #2: In this manuscript, Liu et al report a pipeline that merge two long read technologies and an optical map to produce a gapless assembly of maize chromosome. The data also present the internal structure of major tandem repeats including centromere and heterochromatic knob. I especially look forward to its potential usage in assembling complex genomes with polyploidy or higher levels of heterozygosity.
I have one major concern: I noticed that the only chromosome 3 and 9 are assembled with gapless. Why these two chromosomes are assembled with gapless, rather than other chromosomes? As they are not the shortest chromosome and they are not the chromosome with minimal satellite repeats in maize.

*Answer:
Chromosome 3 and 9 have the least repetitive content compared with other chromosomes. We have now clearly pointed this out in the manuscript.*

Furthermore, did you use your pipeline with the public dataset from maize B73 to assembly the genome. It may be used to evaluate your pipeline with another repeat experiment.

*Answer:
The B73 version 5 genome was assembled separately and involved different methods. While the same PacBio and Bionano methods were used for B73, we did not have Oxford nanopore data for B73, and we did not use the assembly merging pipeline.*

Two minor questions:
1、The author reported that the Cinful-Zeon family of Gypsy elements preferentially target knobs, so how do your think the function of Cinful-Zeon elements in knobs?

*Answer:
It is unlikely that there is any function for Cinful-Zeon in knobs. This transposon is known to also target other heterochromatic regions (as indicated in the manuscript). The very dense heterochromatic nature of knobs may make them more prone to accumulating Cinful-Zeon elements.*

2、The author provided the first complete assembly of mazie Abnomal chromosome 10. The kinesin driver complex binding to knob180 is required for meiotic drive. Whether TR-1 knobs have its own motors encoded on Ab10?

*Answer:
This paper is focused on genome assembly and the structure of major repeats. We have another paper forthcoming that is focused on the genetics, cell biology and biochemistry of the TR-1 motor.*

**Second round of review**

**Reviewer 2**

The authors revised their manuscript accordingly and reply all the questions, now I can make a recommendation  for this paper.
